# Allogeneic Non-Adherent Bone Marrow Cells Facilitate Hematopoietic Recovery but Do Not Lead to Allogeneic Engraftment

**DOI:** 10.1371/journal.pone.0006157

**Published:** 2009-07-07

**Authors:** Stephan Fricke, Manuela Ackermann, Alexandra Stolzing, Christoph Schimmelpfennig, Nadja Hilger, Jutta Jahns, Guido Hildebrandt, Frank Emmrich, Peter Ruschpler, Claudia Pösel, Manja Kamprad, Ulrich Sack

**Affiliations:** 1 Fraunhofer Institute for Cell Therapy and Immunology (IZI), Leipzig, Germany; 2 Institute of Clinical Immunology and Transfusion Medicine, University of Leipzig, Leipzig, Germany; 3 Translational Centre for Regenerative Medicine, University of Leipzig, Leipzig, Germany; 4 Clinic for Radiation Therapy and Radiation Oncology, University of Leipzig, Leipzig, Germany; 5 Clinic for Radiation Therapy, University of Rostock, Rostock, Germany; University of Sheffield, United Kingdom

## Abstract

**Background:**

Non adherent bone marrow derived cells (NA-BMCs) have recently been described to give rise to multiple mesenchymal phenotypes and have an impact in tissue regeneration. Therefore, the effects of murine bone marrow derived NA-BMCs were investigated with regard to engraftment capacities in allogeneic and syngeneic stem cell transplantation using transgenic, human CD4^+^, murine CD4^−/−^, HLA-DR3^+^ mice.

**Methodology/Principal Findings:**

Bone marrow cells were harvested from C57Bl/6 and Balb/c wild-type mice, expanded to NA-BMCs for 4 days and characterized by flow cytometry before transplantation in lethally irradiated recipient mice. Chimerism was detected using flow cytometry for MHC-I (H-2D[b], H-2K[d]), mu/huCD4, and huHLA-DR3). Culturing of bone marrow cells in a dexamethasone containing DMEM medium induced expansion of non adherent cells expressing CD11b, CD45, and CD90. Analysis of the CD45^+^ showed depletion of CD4^+^, CD8^+^, CD19^+^, and CD117^+^ cells. Expanded syngeneic and allogeneic NA-BMCs were transplanted into triple transgenic mice. Syngeneic NA-BMCs protected 83% of mice from death (n = 8, CD4^+^ donor chimerism of 5.8±2.4% [day 40], P<.001). Allogeneic NA-BMCs preserved 62.5% (n = 8) of mice from death without detectable hematopoietic donor chimerism. Transplantation of syngeneic bone marrow cells preserved 100%, transplantation of allogeneic bone marrow cells 33% of mice from death.

**Conclusions/Significance:**

NA-BMCs triggered endogenous hematopoiesis and induced faster recovery compared to bone marrow controls. These findings may be of relevance in the refinement of strategies in the treatment of hematological malignancies.

## Introduction

Today, allogeneic hematopoietic stem cell transplantation (HSCT) is the only curative treatment for many patients with hematological malignancies [1].

Bone marrow [2], peripheral mobilized stem cells [3] and umbilical cord blood [4] are the common sources for HSCT. Despite the use of highly sophisticated therapeutic approaches, HSCT is still associated with a considerable mortality caused by a number of complications such as graft versus host disease (GVHD) [5], infectious diseases [6], veno-occlusive disease [7], donor graft rejection [8], and relapses of the underlying diseases [9]. Therefore, the investigation of alternative therapeutic approaches including the use of new stem cell sources and the optimization of treatment strategies are still in need.

Thus, mesenchymal stem cells (MSCs) are used in clinical hematopoietic stem cell transplantation to support hematopoiesis. Previous studies indicate that co-transplantation of mesenchymal stem cells results in faster engraftment of hematopoietic cells [10], [11]. MSCs as well as MSC derived cells provide growth factors essential for hematopoiesis [12]–[14]. These cells are a very promising stem cell type for transplantation because MSCs are easy available and modest regarding their requirements for in vitro expansion. Furthermore, MSCs show no spontaneous transformations [15] and are characterized as highly adherent fibroblastic cells (counted as CFU-f) [16], [17], which attach within 24 hours [16]. It has also been shown that there are other cells derived from CFU-f, which are able to transform into mesenchymal cells, but also into cells of other lineages [18].

Recent studies have investigated whether non adherent bone marrow cells derived from CFU-f (NA-BMCs) have a therapeutic impact in hematopoietic stem cell transplantation. In contrast to the long expansion time of MSCs that can be a major handicap to cell-based therapies [19], NA-BMCs investigated in this study have a short cultivation time of only 4 days. It has been shown that MSC-like cells within NA-BMCs keep their mesenchymal potential [20] and form CFU-fs in vitro [21], [22], skeletal muscle [23] and bone [24] after in vivo transplantation. NA-BMCs include all cells after cultivation of bone marrow cells. Other results published in the literature indicate controversy regarding the exact identity of cells investigated [20]. These cells could differentiate into hematopoietic as well as stromal cell lineages and were also a major source for adult stem cells [18].

In this work we investigated the effects of allogeneic and syngeneic non adherent bone marrow cells on the reconstitution of hematopoiesis in lethally irradiated CD4−/− C57Bl/6 mice transgenic for human CD4 and HLA-DR3 (triple transgenic mice, TTG) [25], [26]. In this setting the presence of human or murine CD4 after transplantation can be used as a marker for chimerism even in a syngeneic setting. Engraftment of allogeneic and syngeneic stem cells was monitored by murine CD4^+^ cells. Endogenous recovery will lead to expression of human CD4^+^ and/or HLA-DR3^+^ cells.

Here we demonstrate that transplantation of syngeneic NA-BMCs protected 83% of irradiated mice from death. Transplantation of allogeneic NA-BMCs preserved 62.5% of mice from death. Interestingly, in contrast to the syngeneic transplantation procedure, in allogeneic NA-BMCs transplantation no hematopoietic donor chimerism was found, demonstrating that allogeneic NA-BMCs did not engraft but supported endogenous stem cell recovery. Compared to bone marrow controls, hematopoietic regeneration in both NA-BMCs-transplanted groups was faster. These findings might be of relevance in the refinement of strategies in the treatment of hematological malignancies.

## Results

### 1. Determination of lethal irradiation toxicity in triple transgenic mice

To determine the lethal irradiation dose in triple transgenic mice for engraftment of NA-BMCs and bone marrow cells groups of four triple transgenic mice were irradiated with X-rays ranging from 0 to 12 Gy followed by analysis of weight, survival, and recovery of peripheral red blood cells. Irradiation of mice with doses ≥8 Gy led to death of all mice within 19 days, while mice irradiated with doses <8 Gy survived (Figure S1). Irradiated mice served as transplantation controls and received the same injection volume without cells. For further transplantation experiments all mice were irradiated with 8 Gy.

### 2. Characterization of bone marrow cells and NA-BMCs

For characterization of cell subsets, NA-BMCs and bone marrow cells were analyzed by flow cytometry for CD11b, CD117, CD4, CD8, CD19, CD31, CD90, CD117, and MHC-II expression in the CD45^+^ cell fraction.

**Figure 1:**
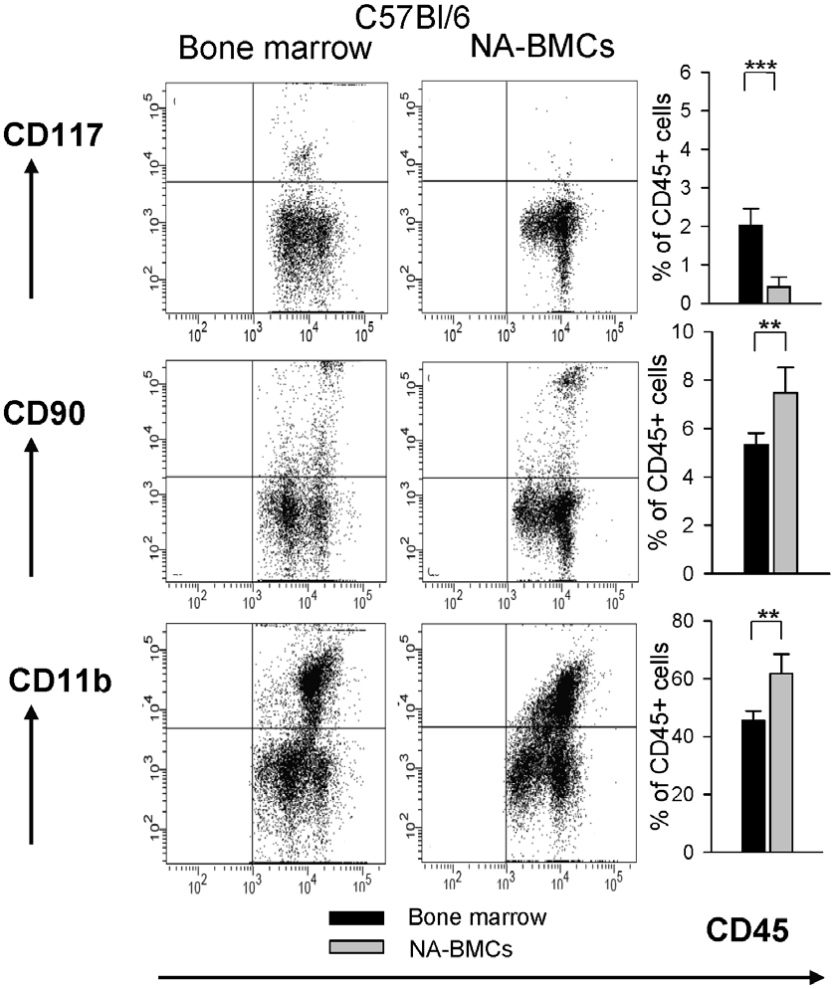
Comparative flow cytometric analysis of bone marrow and NA-BMCs (shown for C57Bl/6). Cells were gated for CD45 expression and co-expression of CD117, CD90 and CD11b for bone marrow cells and NA-BMCs.

Compared to bone marrow cells, NA-BMCs derived from Balb/c and C57Bl/6 showed a significant increase of CD11b^+^ and CD90^+^ and a significant decrease of cells expressing CD117^+^, CD117^+^/CD90^low^, T-cell and B-cell markers (CD4^+^, CD8^+^, CD19^+^) (Figure 1, shown for C57Bl/6 mice and Figure S2). In C57Bl/6 derived bone marrow the proportion of CD45^high^ was 80.5%±0.5% and in Balb/c derived bone marrow 87.4%±2.6%, respectively. After culturing, the proportion of CD45^high^ was 41%±7% in C57Bl/6 mice and 52.7%±14%in Balb/c mice, respectively.

### 3. Transplantation of allogeneic and syngeneic NA-BMCs

For investigation if allogeneic and syngeneic NA-BMCs will engraft after lethal irradiation, triple transgenic mice received either NA-BMCs grafts containing 1×10^5^ to 2×10^6^ NA-BMCs from Balb/c wild-type or C57Bl/6 mice. These experiments were compared to allogeneic and syngeneic bone marrow transplantation using 2×10^6^ cells.

**Figure 2:**
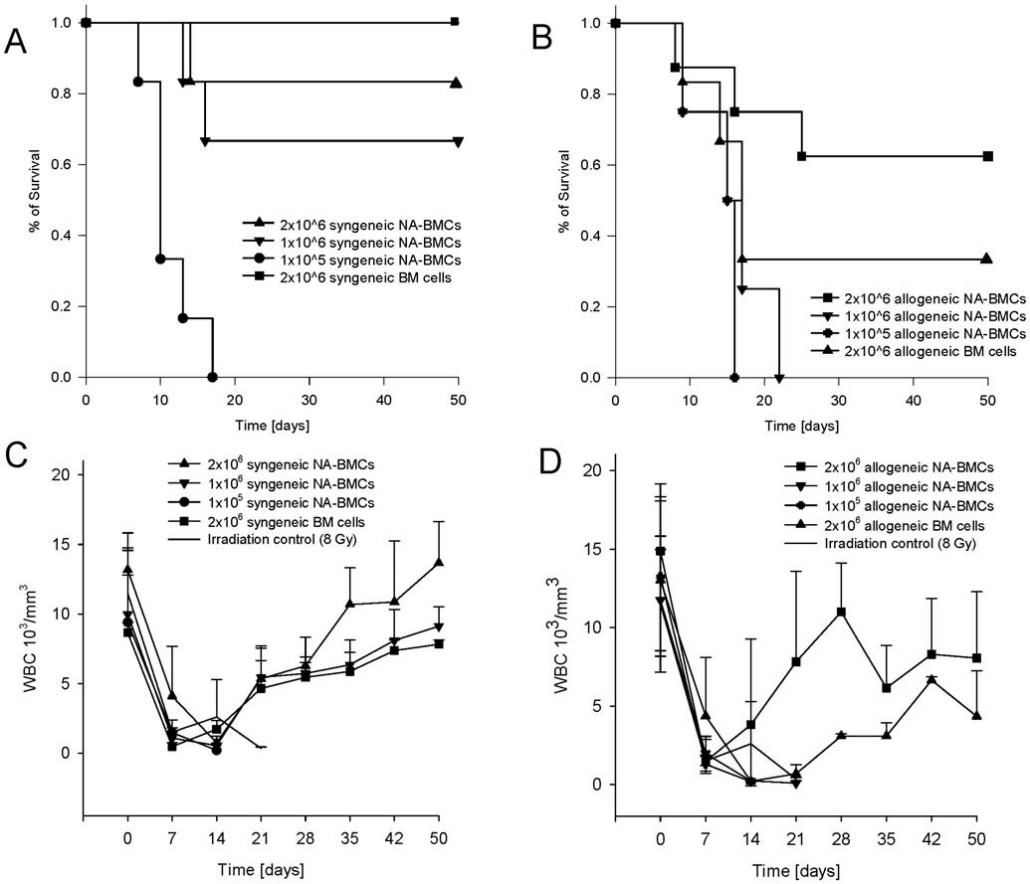
Survival analysis (A+B) and recovery of white blood cell count (WBC, C+D) after irradiation and transplantation. Transgenic mice (C57Bl/6) received either syngeneic or allogeneic NA-BMCs (1×10^5^ to 2×10^6^ cells) or syngeneic and allogeneic bone marrow (2×10^6^ cells). Recovery of WBC after lethal irradiation and transplantation of 2×10^6^ syngeneic NA-BMCs or 2×10^6^ syngeneic bone marrow cells (C) and 2×10^6^ of allogeneic NA-BMCs compared to transplantation of 2×10^6^ allogeneic bone marrow cells (D).

In the control experiments, transplantation of 2×10^6^ syngeneic bone marrow cells preserved 100% of mice from death (Figure 2A). The survival rate of mice that had received 2×10^6^ syngeneic NA-BMCs was 83%, in mice that had received 1×10^6^ it was 66% and 0% in mice receiving 1×10^5^.

Transplantation of 2×10^6^ allogeneic bone marrow cells preserved 33% of mice from death (Figure 2B). Also for ethical reasons we used a system to characterize the clinical status (weight, mobility, texture of the fur, attitude, skin appearance) as published by others [27] for characterization of animals after transplantation. In dying animals, we observed loss of weight, reduced mobility, shaggy fur, and kyphotic attitude. WBC count remained low and time points of death were comparable with the irradiation control groups. Surviving animals showed no signs of GvHD after allogeneic bone marrow transplantation.

In mice that had received 2×10^6^ allogeneic NA-BMCs the survival of mice was 62.5% and 0% in animals that had received 1×10^6^ and 1×10^5^, respectively. Statistical comparisons are given in table 1.

**Table 1:** P values after syngeneic and allogeneic transplantation.

pairs	P values	
	Syngeneic NA-BMCs	allogeneic NA-BMCs
2×10^6^ NA-BMCs vs. 1×10^5^ NA-BMCs	**P = .002	P = .057 NS
2×10^6^ BM cells vs. 1×10^5^ NA-BMCs	**P = .003	P = .094 NS
1×10^6^ naSCs vs. 1×10^5^ NA-BMCs	**P = .007	P = .402 NS
2×10^6^ NA-BMCs vs. 2×10^6^ BM cells	P = .005 NS	P = .5 NS
1×10^6^ NA-BMCs s vs. 2×10^6^ BM cells	P = 0.261 NS	P = .332 NS
and 2×10^6^ NA-BMCs s vs. 1×10^6^ NA-BMCs	P = 0.53 NS	*P = .041

For evaluation of the transplantation procedure related toxicity and engraftment, weekly blood counts in all irradiated mice were performed and concentration of white blood cell count (WBC) was performed (Figure 2C+D). The recovery of WBC after transplantation of 2×10^6^ syngeneic NA-BMCs was significantly earlier at day 35 and 50 compared to transplantation of 2×10^6^ syngeneic BM cells (day 35 *P = .023, day 50 **P = .006). After transplantation of 2×10^6^ allogeneic NA-BMCs, the reconstitution of WBC was also earlier compared to transplantation of 2×10^6^ allogeneic BM cells and achieved significant difference at day 28 (day 28 **P = .019).

**Figure 3:**
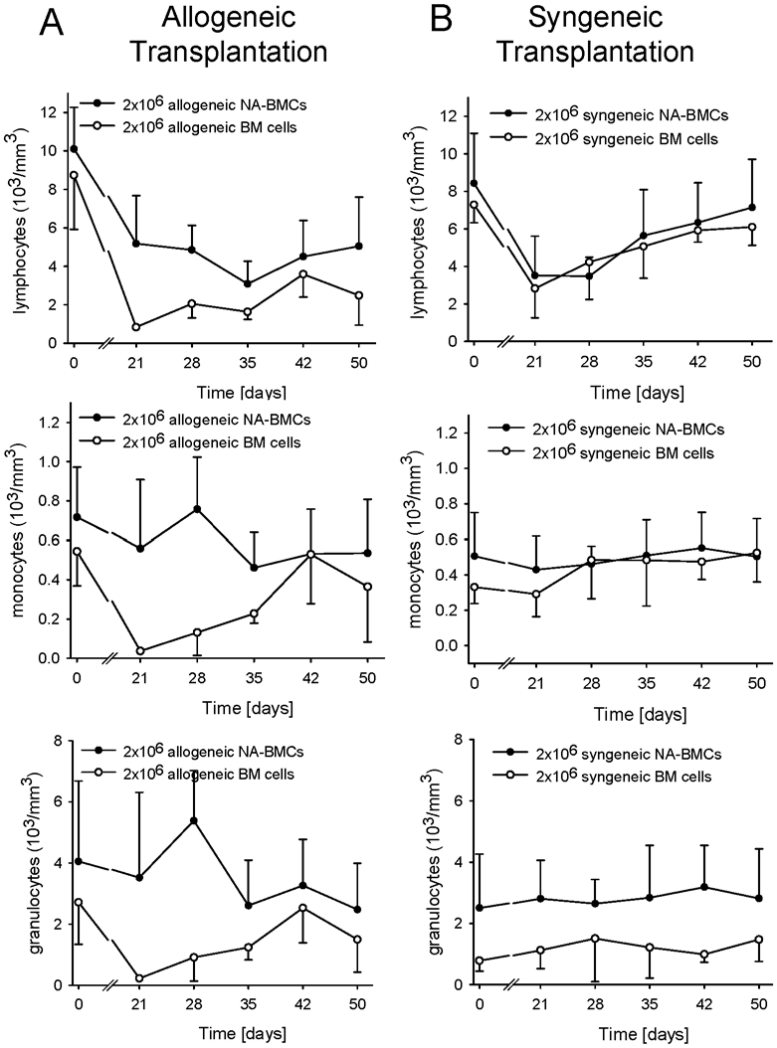
Recovery analysis of lymphocytes, monocytes, and granulocytes after irradiation and transplantation of 2×10^6^ NA-BMCs and 2×10^6^ bone marrow cells. Data are shown for (A) allogeneic transplantation and (B) syngeneic transplantation.

We also determined the distribution of lymphocytes, granulocytes, and monocytes once a week. The recovery of hematopoiesis after allogeneic bone marrow transplantation was delayed for all leukocyte subsets and the lymphocyte subset did not reach the initial levels. After transplantation of 2×10^6^ allogeneic NA-BMCs, granulocytes and monocytes recovered earlier than lymphocytes. The faster WBC recovery after allogeneic NA-BMCs transplantation compared with allogeneic bone marrow transplantation was due to increased myeloid and lymphoid content (Figure 3A).

After syngeneic NA-BMCs and bone marrow transplantation, the reconstitution of hematopoiesis was similar and granulocytes and monocytes recovered earlier than lymphocytes (Figure 3B).

### 4. Analysis of hematopoietic chimerism after transplantation of allogeneic and syngeneic NA-BMCs

**Figure 4:**
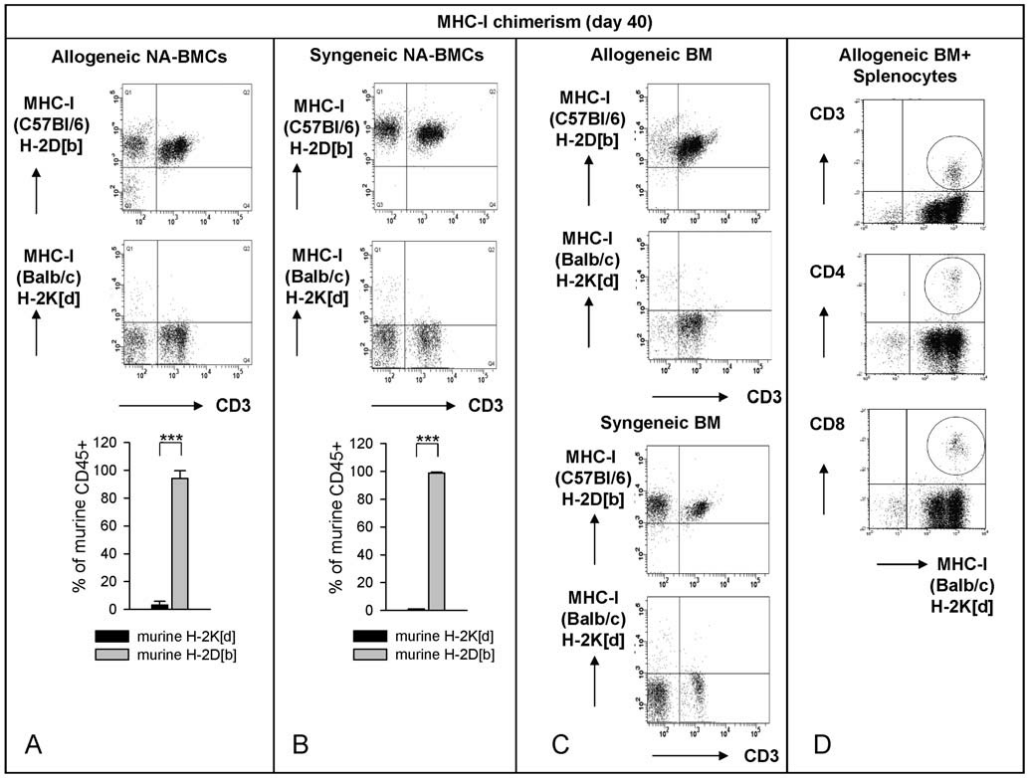
Flow cytometric analysis of MHC-I and CD3 on day -2 before and day 40 after transplantation. MHC-I (H-2D[b], H-2D[d]) and CD3 levels from peripheral blood (gated for lymphocytes) on day 40 after allogeneic NA-BMCs (A), syngeneic NA-BMCs (B) or allogeneic bone marrow (C) transplantation. In co-transplanted animals with allogeneic bone marrow and murine splenocytes we additionally analyze the murine CD4/H-2K[d] and CD8/H-2K[d] expression on bone marrow cells to show donor engraftment.

**Figure 5:**
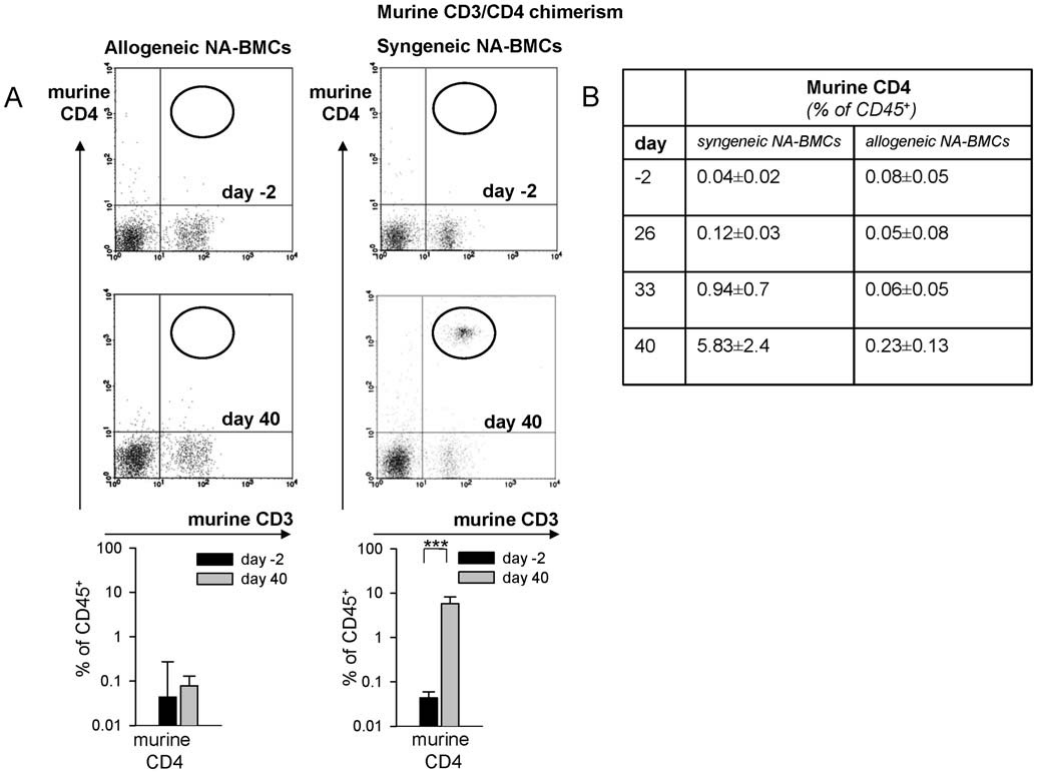
Flow cytometry analysis of murine CD3/CD4 (gated for lymphocytes) from peripheral blood on day -2 before and day 40 after transplantation. Data are shown for transplantation of allogeneic and syngeneic NA-BMCs (A). CD4 molecules were stable expressed after syngeneic transplantation, not after allogeneic transplantation of NA-BMCs (B).

**Figure 6:**
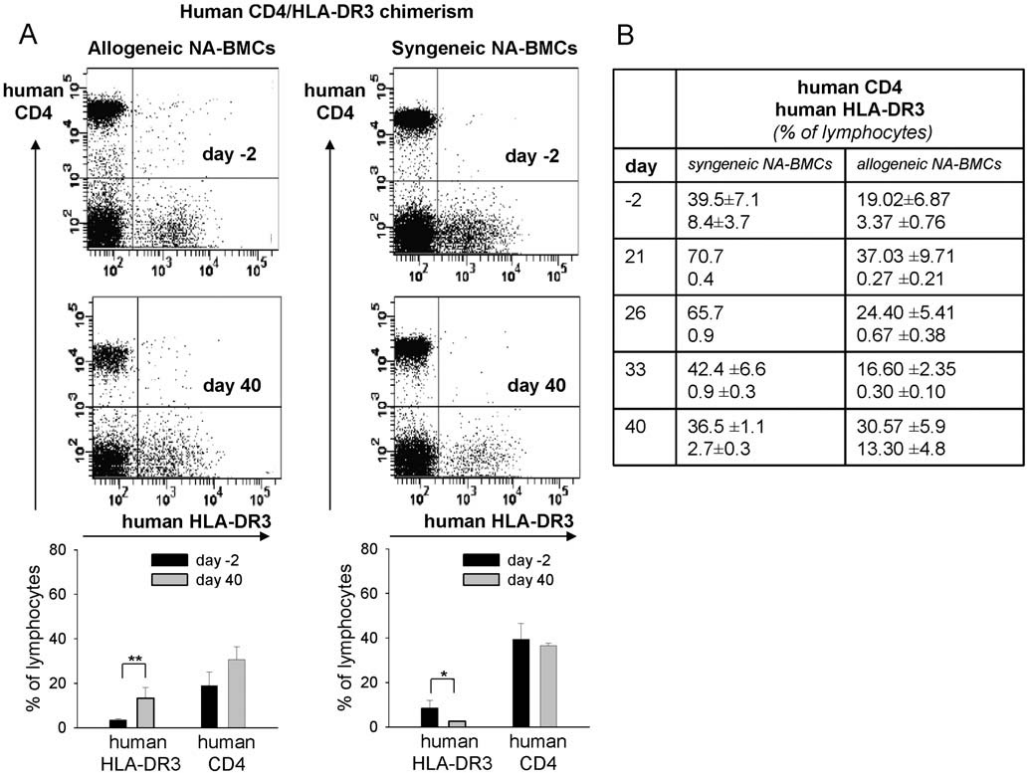
Flow cytometry analysis of human CD4/HLA-DR3 (gated for lymphocytes) from peripheral blood on day -2 before and day 40 after transplantation. Data are shown for transplantation of allogeneic and syngeneic NA-BMCs (A). Human CD4 molecules were stable expressed from day 21 after transplantation until the end of the experiment whereas human HLA-DR3 molecules were not expressed until day 40 (B).

Human and murine CD4 as well as HLA-DR3 molecules were used for detection of chimerism by flow cytometry. On day 40 after transplantation of allogeneic NA-BMCs the MHC-I expression of donor Balb/c (H-2K[d]) remained low (3.1±2.6%) while the recipient MHC-I molecules of C57Bl/6(H-2D[b]) were highly expressed (94.3±5.4%) (Figure 4A). Donor Balb/c (H-2K[d]) MHC-I expression remained low after transplantation until the end of the experiment (Table 2). Murine CD4 was not detectable (0.28±0.13%) (Figure 5A+B). Human CD4 molecules were stable expressed from day 21 until the end of the experiment whereas human HLA-DR3 molecules were not expressed until day 40 (Figure 6A+B) indicating that the recurrence of host HLA-DR3 presenting cells was later than host CD4 T cells.

**Table 2:** Analysis of donor Balb/c (H-2K[d]) chimerism after transplantation of 2×10^6^ allogeneic NA-BMCs.

day	Donor Balb/c (H-2K[d]) *(% of CD45^+^)*
	2×10^6^ allogeneic NA-BMCs
0	1.1±0.4
21	0.1±0.1
33	0.97±0.57
40	3.1±2.62
50	0.57±0.31

After transplantation of syngeneic C57Bl/6 NA-BMCs, recurrence of MHC-I from C57Bl/6 (H-2D[b]) molecule was 98.8±0.8% on day 40 (Figure 4B). Recipient/Donor MHC-I molecules of C57Bl/6 (H-2D[b]) were stable expressed after transplantation (Table 3). Balb/c (H-2K[d]) MHC-I expression remained low and was 1.09±0.40 at day 40 after transplantation. For murine donor CD4, a chimerism of 5.8±2.4% on day 40 was observed (Figure 5A, P<.001) indicating the engraftment of transplanted syngeneic cells. Murine CD4 was detectable for the first time on day 33 and persists until the end of the experiment (Figure 5B). Human CD4 molecules were stable expressed from day 21 until the end of the experiment whereas human HLA-DR3 molecules were not expressed until day 40 (Figure 6A+B). These data indicated the parallel recovery of recipient and donor cells. The recurrence of donor CD4 T cells and host HLA-DR3 presenting cells was later than host CD4 T cells.

**Table 3:** Analysis of donor/recipient C57Bl/6 (H-2D[b]) chimerism after transplantation of 2×10^6^ syngeneic NA-BMCs.

day	Donor/Recipient C57Bl/6 (H-2D[b]) *(% of CD45^+^)*
	2×10^6^ syngeneic NA-BMCs
0	99.19±0.17
21	74.80±12.45
33	97.02±2.56
40	98.55±0.99
50	98.62±0.48

Transplantation of 2×10^6^ allogeneic and syngeneic bone marrow cells induced the reconstitution of a complete host hematopoiesis (Figure 4C). Flow cytometric analysis showed very few non-CD3^+^ lymphocytes and a high proportion of CD3^+^ lymphocytes in surviving mice after allogeneic compared to syngeneic bone marrow transplantation. Co-transplantation of 4×10^6^ allogeneic murine spleen cells and 1×10^7^ allogeneic bone marrow cells induced engraftment of donor cells in surviving mice in this model (Figure 4D).

## Discussion

In this study, we investigated the engraftment kinetics and development of chimerism of allogeneic and syngeneic murine non adherent bone marrow cells (NA-BMCs) transplanted into triple transgenic mice.

The TTG transplantation model allows the analysis of chimerism and the discrimination between donor and host hematopoiesis in syngeneic and allogeneic transplantation settings by the expression of murine or human CD4 and HLA. However, for the detection of non-lymphoid cell lineages other mouse strain combinations will be preferable. For the differentiation of donor and host cells also other congenic mouse strain combinations were used [28]. Using triple transgenic mice, we are able to simulate the functional interaction between human CD4 and human MHC-II molecules. Therefore, the TTG mice will be used for development of GvHD and host versus graft (HvG) treatment strategies as well as for tolerance induction using monoclonal antibodies.

We hypothesized that transplantation of NA-BMCs and bone marrow of C57Bl/6 wild-type mice represents a syngeneic transplantation setting in contrast to bone marrow or NA-BMCs derived from wild type Balb/c representing an allogeneic transplantation setting. However, because multiple mice strains were used for developing the TTG mice, little MHC minor differences can not be totally excluded (for Balb/c MHC-I, H-2K[d] <2% by flow cytometry).

In order to characterize NA-BMC grafts whether they have an impact on HSCT, NA-BMCs were cultured from bone marrow cells by depletion of adherent cells over a period of four days. Flow cytometric analysis showed that CD45 positive NA-BMCs were positive for CD11b, CD90 and had low amounts of CD4^+^, CD8^+^, CD19^+^, and CD117^+^ cells. The amount of hematopoietic stem cells (CD117^+^/CD90^low^) was low. In contrast, bone marrow cells expressed high levels of CD4+, CD8^+^, CD19^+^, and CD117^+^ cells and low levels of CD11b^+^ and CD90^+^ cells.

The proportion of CD45^high^ to CD45^low^ cells was different before and after cultivation of bone marrow cells. During cultivation, the CD45^low^ fraction increased and the CD45^high^ fraction decreased indicating that NA-BMCs contained more progenitors and less differentiated hematopoietic stem cell compared to regular bone marrow cells.

However, there are no direct data demonstrating a true stem cell activity. The term NA-BMCs is not claiming one single cell population. Culturing bone marrow cells over a period of 4 days resulted in a mixed cell population, which probably also contains few HSCs and MSCs. Currently, there are no data that clearly identify a single cell type with simultaneous mesenchymal and hematopoietic capacity. Clonal analyses of the mixed cell culture must be done in future to verify a transplantation effect of a defined cell population.

Next, we investigated the impact and the kinetics of engraftment of syngeneic or allogeneic NA-BMCs on the survival of irradiated TTG mice. Transplantation of syngeneic NA-BMCs preserved up to 83 % of mice in a dose dependent manner. Transplantation of syngeneic bone marrow cells induced hematopoietic recovery of 100% of mice.

Transplantation of 2×10^6^ allogeneic NA-BMCs preserved up to 62.5 % of mice from death. Lower amounts of NA-BMCs did not lead to recovery of hematopoiesis. Transplantation of 2×10^6^ allogeneic bone marrow cells preserved 33% of mice from death, which might indicate that in the TTG model higher numbers of cells with hematopoietic capacity are needed because the additional administration of allogeneic splenocytes induced allogeneic chimerism in surviving mice. Because WBC count remains low and time points of death were comparable with the irradiation control groups in dying animals after allogeneic bone marrow transplantation, we deduced that the cause of death was hematopoietic failure. Surviving animals showed no signs of GvHD after bone marrow transplantation. There are no data available using TTG mice in a bone marrow transplantation setting. The influence of transgenic molecules with regard to mechanisms of engraftment, GvHD, and HvG are still unknown (e.g. distribution and selection of regulatory T cells in transgenic mice). After allogeneic bone marrow transplantation, flow cytometric analysis showed very few non-CD3^+^ lymphocytes and a high proportion of CD3^+^ lymphocytes in surviving mice. Recovery of hematopoiesis was significantly decelerated compared to syngeneic bone marrow or allogeneic/syngeneic NA-BMCs transplantation and did not reach the initial levels. We hypothesize that the immune systems of the surviving recipient mice transplanted with 2×10^6^ allogeneic bone marrow cells are triggered by the graft and endogenous CD3^+^ lymphocytes are activated. This might lead to a shift in CD3^+^ and non CD3^+^ lymphocytes after allogeneic bone marrow transplantation compared to syngeneic bone marrow transplantation.

Interestingly, hematopoietic recovery in mice transplanted with syngeneic or allogeneic NA-BMCs was faster indicated by higher peripheral white blood counts compared to mice that had received syngeneic or allogeneic bone marrow cells.

We also determined the distribution of lymphocytes, granulocytes, and monocytes once a week. After syngeneic and allogeneic NA-BMCs transplantation, the reconstitution of granulocytes and monocytes was earlier than lymphocytes. The recovery of hematopoiesis after allogeneic bone marrow transplantation was delayed for all subsets and did not reach the initial levels. Therefore, the faster WBC recovery after allogeneic NA-BMCs transplantation compared with allogeneic bone marrow transplantation was due to increased myeloid and lymphoid content.

For analysis of hematopoietic chimerism we examined the presence of murine and human CD4 molecules after transplantation of NA-BMCs.

After transplantation of allogeneic and syngeneic NA-BMCs, human CD4^+^ cells that represent the endogenous hematopoiesis were stable expressed from day 21 until the end of the experiment whereas human HLA-DR3^+^ cells on host antigen presenting cells were not detected until day 40. Syngeneic NA-BMCs transplantation led to a recovery of murine CD4^+^ cells not until day 33 after transplantation representing the donor hematopoiesis. The human CD4 molecule is specific for host T cell subsets in this model indicating that recovery of host CD4^+^ T lymphocytes is earlier than recovery of donor CD4^+^ T cells and host HLA-DR3^+^ antigen presenting cells. Therefore, the recovery of endogenous lymphoid cells is earlier than recovery of endogenous antigen presenting cells in this model.

Interestingly, after transplantation of allogeneic NA-BMCs we could detect only human CD4 and HLA molecules as a marker of autologous recovery. By contrast, in the syngeneic setting, chimerism analysis of murine CD4 indicated engraftment of the murine syngeneic donor cells. These data indicate that allogeneic NA-BMCs do not engraft but facilitate autologeous recovery of hematopoiesis.

However, up to now there is no exact mechanism explaining the regulatory effects of allogeneic NA-BMCs. Although, the irradiation dosage is equivalent to the current protocols used in other transplantation settings [29], this minimal dosage probably permits survival of significant numbers of endogenous host stem cells that can be activated after transplantation of allogeneic NA-BMCs. It is possible, that the therapeutic benefit of allogeneic NA-BMCs on improved survival can only be observed using this radiation setting. Therefore, transplantation of NA-BMCs after irradiation with higher dosages should be investigated. As already mentioned above, transplantation of autologeous or allogeneic pluripotent NA-BMCs will reconstitute both the hematopoietic cells and the BM microenvironment [18]. Because NA-BMCs are not a homogenous cell population, remaining MSCs are also able to support host hematopoiesis, e.g. by induction of G-CSF production. The application of G-CSF can also have a supporting effect after lethal irradiation. The effects of recombinant canine granulocyte colony-stimulating factor (rcG-CSF) and recombinant canine stem cell factor (rcSCF) on the circulation of hematopoietic progenitor and stem cells were studied in a canine model [30]. In this study, control animals transplanted with 1×10^8^ PBMC/kg collected without pre-treatment died with marrow aplasia after irradiation as did animals treated with only low-dose SCF before cell collection. In contrast, all animals given PBMC collected after G-CSF, high-dose SCF, or a combination of G-CSF plus low-dose SCF recovered granulocyte function. It has been shown that use of supportive care and G-CSF enhanced recovery of myelopoiesis and survival after lethal doses of irradiation [31] although the authors postulate that administration of G-CSF should be as soon as possible after irradiation. Other investigators have demonstrated that administration of HGFs (GM-CSF, IL-3 and SCF) in lethally irradiated C3H mice did not convey any *in vivo* radioprotection [32].

Other groups showed that stromal cells derived from hematopoietic progenitors (probably present in NA-BMCs) support hematopoiesis [33]. There are several studies in animal models demonstrating that stromal cells not only seed the bone marrow but also enhance hematopoietic recovery [34], [35]. The differentiation of single BM derived cell types along both hematopoietic and mesenchymal lineages is possible and controversially discussed [20].

Our findings show that NA-BMCs engraft in a syngeneic transplantation setting and lead to survival of at least fifty days. Therefore, these cells have supportive function for hematopoiesis. In the allogeneic transplantation setting NA-BMCs support autologous recovery indicated by detection of host chimerism. Syngeneic and allogeneic NA-BMCs induce faster recovery of hematopoiesis and reduce transplantation related toxicity. To our knowledge this is the first report describing such effects of NA-BMCs in mice. These findings may have an impact on clinical transplantation settings. NA-BMCs will probably have an impact in treatment of critically ill hematological patients after allogeneic transplantation. Furthermore, NA-BMCs might be useful in cases of severe irradiation exposure or toxic organ damage. An important finding is the higher frequency of relapses in leukemia patients after allogeneic bone marrow transplantation compared to peripheral mobilized blood stem cell grafts [3]. NA-BMCs can not only trigger stem cells of the graft (as shown for murine CD4) but also trigger residual hematopoietic stem cells of the host (as shown for human CD4 and HLA-DR3). Therefore, NA-BMCs may be able to trigger remaining tumor cells that probably reduce the therapeutic applicability in leukemic patients. This could be caused by higher frequencies of progenitors in bone marrow derived NA-BMCs compared to peripheral blood. However, the effect of NA-BMCs regarding tumor recurrence and graft versus tumor effect must be investigated in appropriate tumor models.

## Materials and Methods

### Ethics Statement

All mice were housed, treated or handled in accordance with the guidelines of the University of Leipzig Animal Care Committee and the Regional Board of Animal Care for Leipzig (animal experiment registration number 24/06).

### Animals

C57Bl/6 CD4k/o mice transgenic for human CD4 and HLA-DR (TTG mice) were bred at the Animal Facility at the University of Leipzig. The mice strain was maintained under standardized conditions. These mice have a stable C57Bl/6 background, in which the murine CD4 molecule is knocked out and replaced by human CD4. The CD4 transgene includes its own promoter ligated to a murine CD4 enhancer element thus leading to T cell subset-specific expression. CD8^+^ cells are not affected in TTG mice Furthermore, these mice express the HLA-DR3 molecule in addition to the murine MHC II complex. The TTG mice have complete functional murine immune system which is modified with regard to CD4 and HLA-DR [36]. The mice were fed ad libitum. As donors, C57Bl/6 and Balb/c mice were purchased from Charles River (Sulzfeld, Germany; http://jaxmice.jax.org).

### Irradiation protocol

For irradiation of mice the X-Ray apparatus (D3225, Orthovoltage, Gulmay Medical, Camberley, UK) was adjusted for animal irradiation. 4 animals were irradiated in parallel in a plexiglass container (divided in five spaces per 0.5 cm×4.0 cm). For determination of the lethal irradiation dose, 16 mice underwent single total body irradiation with irradiation doses from 3 Gy to 12 Gy (200 kV, dose rate 1,14 Gy/min), 4 mice were used as non irradiated control mice.

### Culture of non adherent bone marrow cells (NA-BMCs) derived from murine bone marrow and preparation of bone marrow and splenocytes

Bone marrow cells (BMCs) were obtained from tibiae and femura from C57Bl/6 and Balb/c wild-type mice according to the method of Dobson et al [37], and expansion was started at a concentration of 2×10^6^/55 cm2 Petri dishes in 10 ml Dulbecco's modified Eagle's minimal essential medium (DMEM, Perbio, Bonn, Germany) modified as described by others [20]. The medium was supplemented with 10% fetal calf serum (FCS, Invitrogen, Karlsruhe, Germany) and dexamethasone (10^−8^ M). Murine splenocytes were prepared as described elsewhere [38].

### Flow cytometry

#### Characterization of murine NA-BMCs and bone marrow cells

For cytometric analysis, 2×10^5^ cells were incubated with 2.5 µl of conjugated monoclonal antibodies (CD3-FITC, CD8-APC [both Beckman Coulter, Krefeld, Germany]; MHC-II-PE, CD4-PECy7, CD45-PerCP, CD19-APCCy7, CD31-FITC, CD90-PE, CD117-APCCy7, CD11b-APCCy7 [BD Biosciences, Heidelberg, Germany]). A 25 minutes incubation was followed by two washing steps in PBS/1% FBS (1250 rpm, 5 minutes, RT). Finally the pellet was resuspended with 200 µl of PBS/3% formaldehyde (Merck, Darmstadt, Germany). Data were acquired on a BD FACSCantoII™ Flow Cytometer and analysed using the BD FACSDIVA™ software (both BD Biosciences, Heidelberg, Germany).

#### Flow cytometry and hematology of TTG mice

Before and after transplantation procedure, recipient mice were analyzed by flow cytometry. At particular time points, blood (150 µl) was taken from the retro orbital vein of each mouse under ether anesthesia. Blood was collected through heparinized capillaries (Greiner Biochemica, Flacht, Germany). Hemoglobin concentration was determined using an Animal Blood Counter (SCIL, Viernheim, Germany), which had been calibrated for mouse blood within 2 hours after blood taking.

For cytometric analysis 100 µl of blood cells were incubated with 2.5 µl of conjugated monoclonal antibodies according to samples (murine CD4-PE, MHC-I (H-2D[b])-PE, MHC-I (H-2K[d])-PE [BD Biosciences, Heidelberg, Germany]; murine CD3-FITC, murine CD8- APC, human CD4-PE [Beckman Coulter, Krefeld, Germany]; human HLA-DR3-FITC [Immunotools, Friesoythe, Germany]). 25 minutes incubation was followed by erythrocyte lysing according to manufacturers instructions (BD FACS Lysing Solution [BD Biosciences, Heidelberg, Germany]). By adding of PBS/1% FBS samples were washed twice (1250 rpm, 5 minutes, room temperature [RT]). Finally, the pellet was resuspended with 200 µl of PBS/3% formaldehyde (Merck, Darmstadt, Germany). Data were acquired on a BD FACSCantoII™ Flow Cytometer and analysed using the BD FACSDIVA™ software (both BD Biosciences, Heidelberg, Germany).

### Cell Transplantation

The NA-BMCs transferred from one dish into a new dish for 3 days and non adherent cells were harvested at day four, washed in PBS and pelleted. After that, 3 wash steps with normal saline (0.9% NaCl) followed. Murine splenocytes were used freshly after preparation. Finally, the cell concentration was adjusted to 2×10^6^–1×10^7^ cells per 150 µl of sterile 0.9% NaCl. For co-transplantation experiments, 4×10^6^ of allogeneic murine splenocytes were added to 1×10^7^ of allogeneic bone marrow cells. The grafts were subsequently injected intravenously within the next 7 hours into the lateral tail vein of lethally irradiated recipient mice. Engraftment of cells was investigated for 50 days.

### Statistical Analysis

All data are presented as means±SD. Non-linear regression models for describing the time course of hematological parameters as a function of dose and time were developed by Mr. Thomas Keller, PhD (ACOMED statistics, Leipzig, Germany) by using SPSS 15.0 software package (SPSS Science, Erkrath, Germany, supplementary material 1). Statistic analysis and graphic presentation were made using SigmaPlot 10.0/SigmaStat 3.5 software (SYSTAT, Erkrath, Germany).

## Supporting Information

Figure S1 Development of non-linear regression models of function parameter = function (dose, time) for red blood cell count (RBC) and determination of the lethal irradiation dose. (A) Time was modeled using exponential decay and sigmoidal increase or decay. Both functions are related to typical damage processes and growth processes, respectively. (B) Groups of four mice were irradiated with X-Rays (0 Gy, 3 Gy, 6 Gy, 8 Gy, 12 Gy). Survival for doses ≥8 Gy is significantly shorter than for doses <8 Gy.
(0.04 MB PDF)

Figure S2 Complete comparative flow cytometric analysis of bone marrow and NA-BMCs from Balb/c and C57Bl/6. Cells were gated for CD45 expression and co-expression of CD4, CD8, CD19, CD117, CD117^+^/CD90^low^, CD31, CD11b, MHC-II, CD90 for bone marrow cells and NA-BMCs derived from Balb/c and C57Bl/6.
(0.01 MB PDF)
