# Correction: Allogeneic Non-Adherent Bone Marrow Cells Facilitate Hematopoietic Recovery but Do Not Lead to Allogeneic Engraftment

**DOI:** 10.1371/journal.pone.0136005

**Published:** 2015-08-14

**Authors:** Stephan Fricke, Manuela Ackermann, Alexandra Stolzing, Christoph Schimmelpfennig, Nadja Hilger, Jutta Jahns, Guido Hildebrandt, Frank Emmrich, Peter Ruschpler, Claudia Pösel, Manja Kamprad, Ulrich Sack

The authors wish to provide clarifications and corrections to some inaccuracies in the published paper. Incomplete data was reported for the number of mice in each experimental group. The n for each group is provided in the following revised figure legend for Fig 2, along with clarification that 1.0 on the y-axis corresponds to 100% survival:

**Fig 2:**
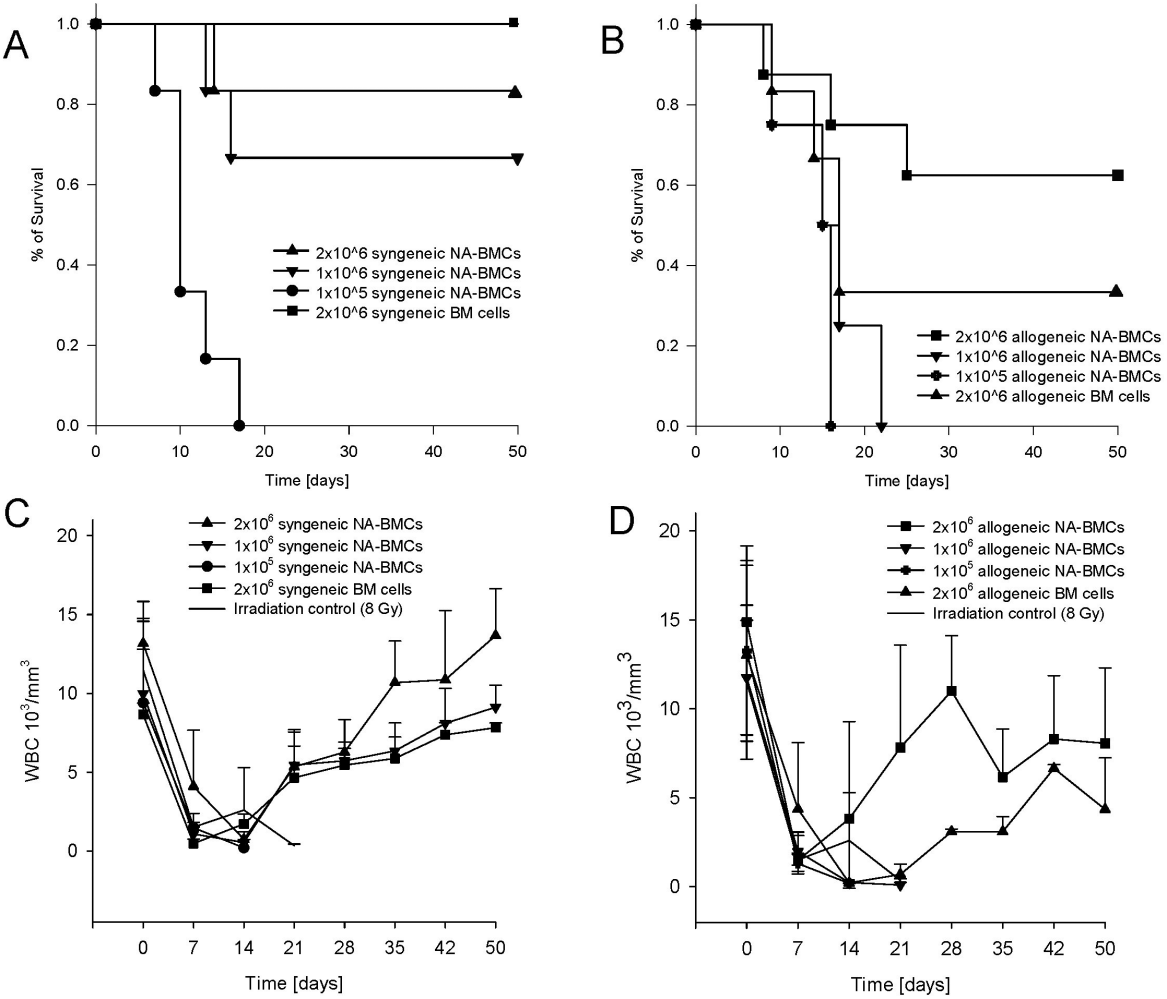
Survival analysis (survival 1.0 = 100%) (A+B) and recovery of white blood cell count (WBC, C+D) after irradiation and transplantation. Transgenic mice (C57Bl/6) received either syngeneic or allogeneic NA-BMCs (1 x 105 to 2 x 106 cells) or syngeneic and allogeneic bone marrow (2x106 cells). Recovery of WBC after lethal irradiation and transplantation of 2x106 syngeneic NA-BMCs or 2x106 syngeneic bone marrow cells (C) and 2x106 of allogeneic NA-BMCs compared to transplantation of 2x106 allogeneic bone marrow cells (D). Syngeneic groups 1x105 to 2x106 NA-BMCs n = 6, group 2x106 BM cells n = 4. Allogeneic groups 1x106 and 1x105 NA-BMCs n = 4, group 2x106 NA-BMCs n = 8, group 2x106 BM cells n = 6.

There was an error in statistical comparisons for the survival study due to the use of incorrect data on the number of mice in some groups. When the correct n-numbers for each experimental group are considered, the P values of Table 1 should be revised. Additionally, NA-BMCs are mislabelled as “naSCs” in one entry of the table. A corrected Table 1 is provided here.

**Table 1:** P values after syngeneic and allogeneic transplantation.

	P values	
Pairs	Syngeneic NA-BMCs	Allogeneic NA-BMCs
2 x 10^6^ NA-BMCs vs. 1 x 10^5^ NA-BMCs	**P = .002	*P = .023
2 x 10^6^ BM cells vs. 1 x 10^5^ NA-BMCs	**P = .003	P = .124 NS
1 x 10^6^ NA-BMCs vs. 1 x 10^5^ NA-BMCs	**P = .007	P = .354 NS
2 x 10^6^ NA-BMCs vs. 2 x 10^6^ BM cells	P = .41 NS	P = .309 NS
1 x 10^6^ NA-BMCs s vs. 2 x 10^6^ BM cells	P = .23 NS	P = .39 NS
and 2 x 10^6^ NA-BMCs s vs. 1 x 10^6^ NA-BMCs	P = .53 NS	*P = .026

We also provide a revised sentence for the Abstract with corrected n as follows: “Syngeneic NA-BMCs protected 83% of mice from death (n = 6, CD4+ donor chimerism of 5.8±2.4% [day 40], P < .001).”

All other data analysis employed the correct n numbers and is unaffected by this correction.

We also wish to clarify the statistical tests used to analyse the data by adding the following sentence to the Methods section on Statistical Analysis: “Analysis of survival curves was done using the log-rank test, analysis of other parameters with t-tests, the Mann–Whitney U test or the Holm-Sidak test.”

Finally, we provide additional information regarding the survival study as follows: mice were humanely euthanized by CO2 inhalation upon meeting certain criteria based on weight, mobility, texture of the fur, attitude, and skin appearance.

We apologize for any inconvenience resulting from the errors. These corrections do not change any conclusions or statements that are given in the publication.
