# Supplementary figures and images for: Allogeneic Non-Adherent Bone Marrow Cells Facilitate Hematopoietic Recovery but Do Not Lead to Allogeneic Engraftment
Source: PLoS One. 2009 Jul 7;4(7):e6157. doi: 10.1371/journal.pone.0006157 (PMC2701999; doi:10.1371/journal.pone.0006157)

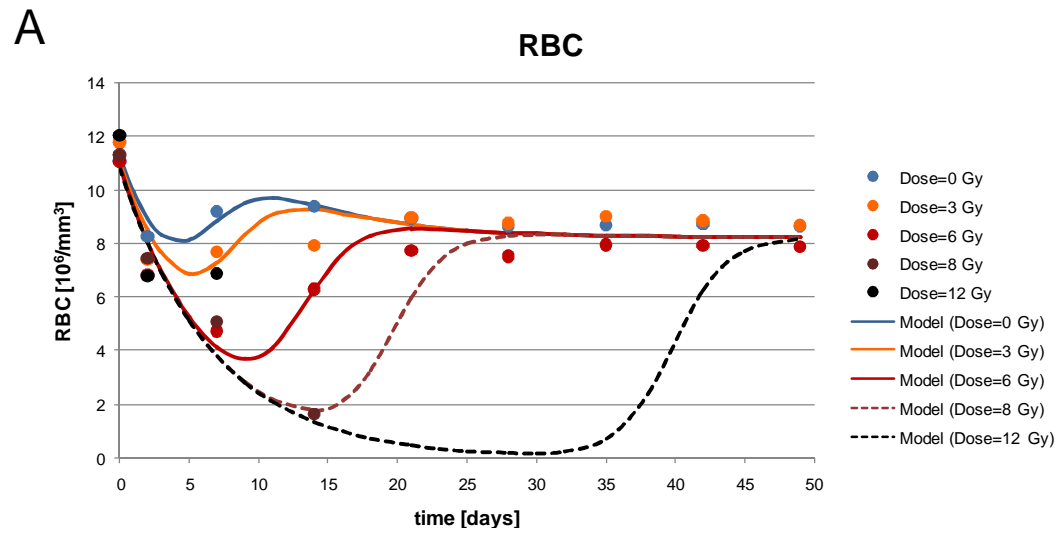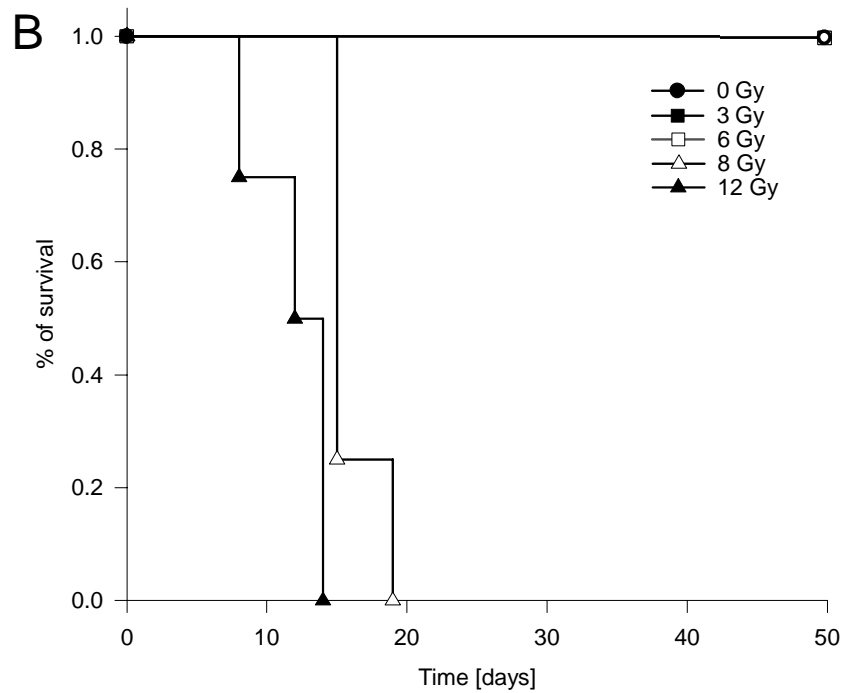

Supplement: Figure S1 — Development of non-linear regression models of function parameter = function (dose, time) for red blood cell count (RBC) and determination of the lethal irradiation dose. (A) Time was modeled using exponential decay and sigmoidal increase or decay. Both functions are related to typical damage processes and growth processes, respectively. (B) Groups of four mice were irradiated with X-Rays (0 Gy, 3 Gy, 6 Gy, 8 Gy, 12 Gy). Survival for doses ≥8 Gy is significantly shorter than for doses <8 Gy. (0.04 MB PDF) [file pone.0006157.s001.pdf]

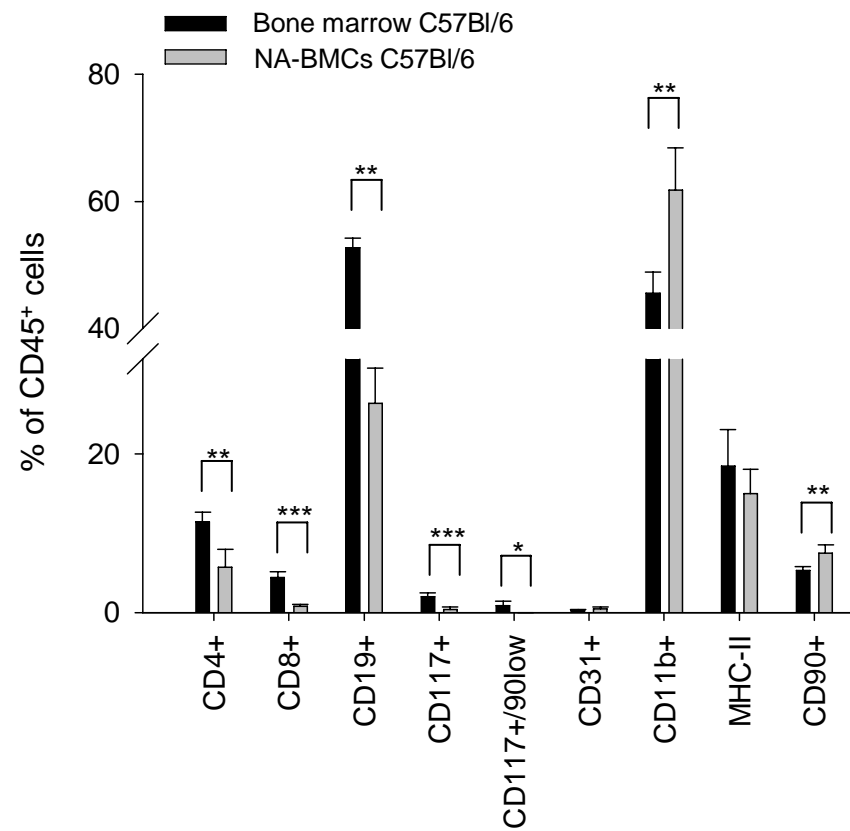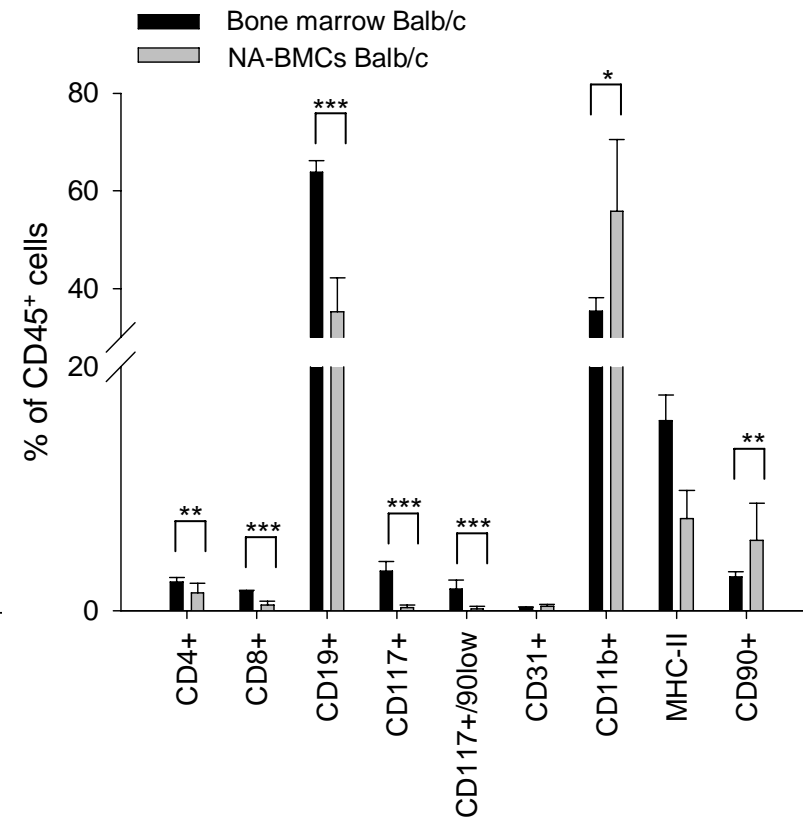

Supplement: Figure S2 — Complete comparative flow cytometric analysis of bone marrow and NA-BMCs from Balb/c and C57Bl/6. Cells were gated for CD45 expression and co-expression of CD4, CD8, CD19, CD117, CD117+/CD90low, CD31, CD11b, MHC-II, CD90 for bone marrow cells and NA-BMCs derived from Balb/c and C57Bl/6. (0.01 MB PDF) [file pone.0006157.s002.pdf]
